# Eating Behaviors among Online Learning Undergraduates during the COVID-19 Pandemic

**DOI:** 10.3390/ijerph182312820

**Published:** 2021-12-05

**Authors:** Christine Yeong Ying Pung, Seok Tyug Tan, Seok Shin Tan, Chin Xuan Tan

**Affiliations:** 1 Department of Allied Health Sciences, Faculty of Science, Universiti Tunku Abdul Rahman, Jalan Universiti Bandar Barat, Kampar 31900, Perak, Malaysia; cpyying@gmail.com; 2 Department of Healthcare Professional, Faculty of Health and Life Sciences, Management and Science University, University Drive, Off Persiaran Olahraga, Seksyen 13, Shah Alam 40100, Selangor, Malaysia; sttan@msu.edu.my; 3 Department of Nutrition and Dietetics, School of Health Sciences, International Medical University, Bukit Jalil, Kuala Lumpur 57000, Malaysia; seokshin_tan@imu.edu.my

**Keywords:** COVID-19, eating behaviors, online learning

## Abstract

The outbreak of Coronavirus Disease-2019 (COVID-19) has upended education systems. The pandemic switched traditional face-to-face classes to online classes. Poor eating behaviors in undergraduate students’ lives may affect the well-being of their later stages of life. This study aimed to investigate the eating behaviors among online learning undergraduates during the COVID-19 pandemic. A total of 122 students were recruited. The results revealed more than half of the respondents (52.46%) skipped meals. Breakfast (46.72%) was the most skipped meal. The majority of the respondents (94.26%) snacked between meals. Biscuits (88.52%), bread (84.43%), and fruits (80.33%) were the most common snacked foods. Meal skipping was found to be associated with gender (*χ*^2^ = 6.83, *p* < 0.05). In the future, more health interventions program aimed to promote healthy eating behaviors among undergraduates are highly warranted.

## 1. Introduction

Coronavirus Disease-2019 (COVID-19) is caused by a virus known as severe acute respiratory syndrome coronavirus 2 (SARS-CoV-2). It was first discovered in Wuhan, China in December 2019 and declared as a pandemic by World Health Organization (WHO) in March 2020 [1]. This pandemic had led to 5,127,696 death cases globally and 29,892 death cases in Malaysia as of 19 November 2021 [2]. In view of the rising number of active and death cases, the government of Malaysia ordered the closure of universities, cancellation of all physical classes, and transition to online learning mode under the Movement Control Order (MCO). This is because the SARS-CoV-2 virus can be easily transmitted when the infected person coughs or sneezes. To prevent the spread of the virus by practicing physical distancing, the Malaysian education system implemented online teaching and learning.

Eating behavior is a widely used term that includes preferences of food and intentions, eating habits, dieting, and problems that are related to eating, such as feeding disorders, eating disorders, and underweight or overweight [3], while meal skipping is defined as the omission of one or more main meals throughout the day [4]. Young adults aged 18–30 years in the period of transition and development might possess higher rates of meal skipping [4].

The study of Gan et al. [5] showed that the majority of the Malaysian undergraduates skipped at least one meal daily in the past seven days. Common reasons for meal skipping were reported to be insufficient time, price of food, and weight maintenance [4]. Another study conducted in Malaysia revealed that most undergraduates (73.5%) from medical science have low consumption of fruits, i.e., not more than three times in a week, and 51.5% of them consumed fried food for the frequency of twice or more a week [6]. According to Gallo et al. [7], the energy intake by female university students during the COVID-19 pandemic was 20% greater than the usual intake. This study even showed a rise in the snacking frequency during this COVID-19 pandemic, especially in individuals with a BMI higher than the normal range [8].

The teaching and learning in Malaysia’s education system changed from face-to-face to online mode since the COVID-19 outbreak in March 2020. A recent study reported that eating behaviors during home confinement was unhealthier [9,10]. Currently, there are few studies available that examined the eating behaviors among online learning undergraduates during the COVID-19 pandemic in Malaysia. Hence, this study aimed to determine the eating behaviors among online learning undergraduates during the COVID-19 pandemic.

## 2. Material and Methods

This cross-sectional study was conducted using a questionnaire created through Google Forms. Data collection was carried out in the period of 20 January 2021 to 25 February 2021 using a convenience sampling approach. Online platforms (Microsoft Teams, email, and WhatsApp) were utilized to circulate the questionnaire to the online learning undergraduates studied in a private university in Kampar, Perak, Malaysia. The sample size was calculated using Cochran’s formula with a prevalence rate of 6% (Prevalence of university students with abnormal eating behaviors reported by Abdalla et al. [11]), a normal deviation at 95% confidence interval, and ±5% error of precision. After adjusting 10% of the non-response rate, the minimum sample size required was 96; a total of 122 respondents who met the inclusion criteria of being Malaysian, undergraduate, multi-racial, and multi-ethnic were recruited. The exclusion criteria were postgraduate, on medication, and the presence of systematic disease. Approval from this study was obtained from Universiti Tunku Abdul Rahman Scientific and Ethical Review Committee with the ethical code Re: U/SERC/23/2021.

### 2.1. Assessment of Eating Behaviors

An online questionnaire consisting of informed consent, sociodemographic information, and Eating Behaviors Questionnaires (EBQ) was used. Respondents who gave consent to voluntarily participate in the present study would select the ‘Agree’ option and would then be directed to complete the online questionnaire. In the sociodemographic segment, questions on gender, ethnicity, age, current living arrangement, academic year, height (centimeter), and weight (kilograms) were gathered. Respondents were required to self-report their weight and height. The nutritional status of the respondents was estimated using the body mass index (BMI) according to the cut-off points of WHO standards [12]. The EBQ was a validated instrument developed by Chin and Nasir [13] to assess eating behaviors. This questionnaire consisted of questions on assessing the frequency of meal intake, frequency of snack intake between meals, types of snack foods and beverages intake, frequency of eating away from home, use of health supplements, dietary practices, and participation in weight control programs. The questionnaire of this study is accessible upon email request to the corresponding author. 

### 2.2. Statistical Analysis

IBM SPSS Statistics 26 (IBM Corp, Armonk, NY, USA) was used to analyze the data. Descriptive statistics such as mean, standard deviation (SD), frequency, and percentage were used to describe all variables. Chi-squared test of independence was used to estimate associations between sociodemographic variables (gender, age, current living arrangement, academic year, and BMI) and meal skipping behavior. The statistical significance was set at *p* < 0.05.

## 3. Results

### 3.1. Socio-Demographic Information

Table 1 shows the socio-demographic information of the respondents. Most of the respondents were female (*n* = 100, 81.97%), Chinese (*n* = 116, 95.08%), aged 20–22 years old (*n* = 106, 86.89%), year 3 to 4 students (*n* = 72, 59.02%), staying with family members (*n* = 101, 82.79%), and had a normal BMI value (*n* = 72, 59.02%).

### 3.2. Meal Consumption and Meal Skipping Behaviors

**Figure 1:**
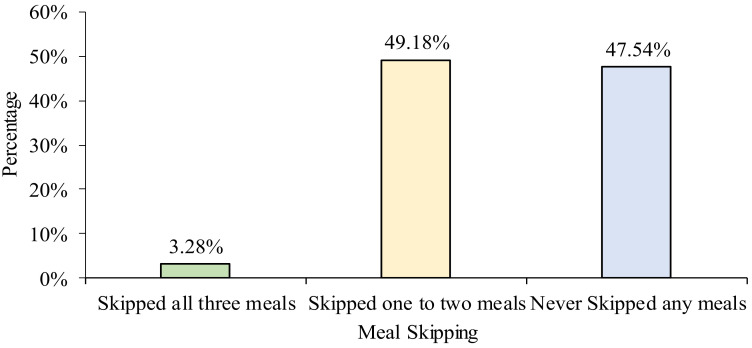
Frequency of meal skipping.

**Figure 2:**
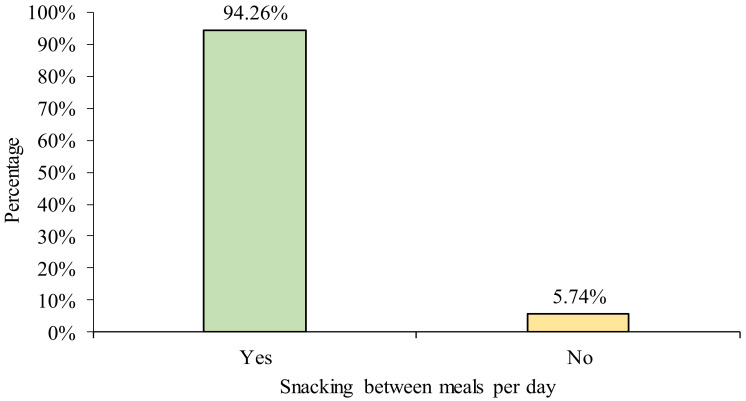
Snacking behavior of the participants.

Table 2 shows the frequency of main meal intake and snacking behavior among online learning undergraduates. The majority of the respondents (90.16%) consumed dinner every day and more than half of the respondents (53.28%) consumed breakfast daily. Apart from main meals, 82.79% and 72.95% of the respondents snacked during afternoon tea and supper, respectively. Figure 1 and Figure 2 summarize the meal skipping and snacking behaviors of the respondents in this study. Almost half of the respondents (49.18%) skipped one to two meals in a day, 47.54% did not skip any of the main meals, while the minority (3.28%) skipped breakfast, lunch, and dinner. Meanwhile, most of the respondents (92.26%) consumed snacks between meals. Biscuits (88.52%), bread (84.43%), and fruits (80.33%) were the most commonly consumed foods between meals (Table 3), whereas milk (66.39%), tea (58.20%), and chocolate malt drink (49.18%) were the most frequently consumed beverages between meals (Table 4).

### 3.3. Eating Away from Home

Table 5 shows the frequency of eating out among the respondents. The majority of the respondents ate out at hawker centers, coffee shops or other food stalls (27.05%), and Western fast-food restaurants (45.08%) one to three times per month. 

### 3.4. Dietary Practices

Table 6 shows the dietary practices of respondents while online learning during the COVID-19 pandemic. A higher percentage of the respondents (36.07%) consumed any available food, while 19.67% limited the intake of high-fat and high-sugar foods, and 16.39% did not eat according to any special diet menu but just reduced the food intake to prevent weight gain. However, 12.30% of the respondents practiced low intake of high-fat, high-sugar, and red meat foods. The rest (9.02%) practice other diets, such as less sodium, fewer carbohydrates, and a high-protein diet.

### 3.5. Health Supplements Intake

Table 7 shows health supplements consumption and the sources of advice on health supplement consumption among online learning undergraduates. More than half of the respondents (59.02%) did not consume supplements, while 40.98% consumed supplements. Among those consumed supplements, 20.49% consumed based on parents’ advice, 11.48% consumed based on their own decision, while 4.92% consumed based on information from websites or social media, 2.46% eat according to the advice from other family members, and 1.64% consumed due to a friend’s advice. None of them consumed according to the advice from physicians.

### 3.6. Participation of Weight Control Program 

The participation in weight control program among the online learning undergraduates is shown in Table 8. The majority of the respondents (95.90%) did not join a weight change program while only 4.1% did join one. Among the respondents who joined, 1.64% joined gyms and sports, respectively, whereas 0.82% joined yoga. Sources of joining the weight change program were parents’ advice (1.64%), social media or websites (1.64%), and own decision (0.82%).

### 3.7. Association between Sociodemographic Variables and Meal Skipping Behaviors

The association between sociodemographic variables and meal skipping behaviors is tabulated in Table 9. Gender was found to be associated with meal skipping (*χ*^2^ = 6.83, *p* < 0.05), while other variables (age, academic year, living arrangement, and BMI) were not associated with meal skipping behaviors.

## 4. Discussion

This study demonstrated that majority of the online learning undergraduates had normal BMI (>50%) and the proportion of respondents with underweight (27%) were found to be higher than overweight and obese (14%) students. These results were similar to previous studies done during the non-COVID-19 period [14,15]. According to Ren et al. [16], the increasing trend of underweight among university students might be due to insufficient nutrient intake as a result of poor eating behavior.

Globally, unhealthier food intake and eating habits were observed during the COVID-19 home confinement [9]. Our current study revealed that more than half of the online learning undergraduates practiced meal skipping behaviors. A study published by Son et al. [17] among college students in the United States revealed that 20% of the surveyed respondents had inconsistent eating patterns such as irregular meal intake and skipped meals during the COVID-19 pandemic. The reasons behind meal skipping during the COVID-19 pandemic may be due to the low accessibility to purchase food from physical stores [18]. It was reported that meal skipping in young adults was associated with the increment of cardiometabolic risks in their later life [19]. 

More than 90% of the online learning undergraduates consumed snacks during the COVID-19 pandemic. Current study revealed the most popular snack foods were biscuits (88.52%), bread (84.43%), and fruits (80.33%). Generally, undergraduates that live with family members tend to increase the intake of fruits as compared to undergraduates who live alone or far away from their family members [20]. It was found that students tend to snack while watching TV or studying [21]. Boredom is one of the reasons to add snacks into daily diet among university students [18]. In addition, students with higher stress levels tend to practice unhealthy eating behavior such as consumption of sugary snacks, food that is rich in carbohydrates content, junk food, and ready-to-eat processed food [22].

Furthermore, more than half (>50%) of the surveyed undergraduates had their meals with family members. Students that live with their family members have a high tendency to consume home-cooked meals rather than dining in at restaurants [23]. In Malaysia, the COVID-19 outbreak resulted in the transition of the physical lectures in universities into virtual lectures [24,25]. Hence, many undergraduates may have returned to their homes and stayed with their family members. This results in passive roles in food selections [18].

Our study also revealed that 41% of the online learning undergraduates consumed health supplements, and parents were found to be their main adviser in taking the supplements. A same phenomenon was reported by Choi [26], in which the majority (49.7%) of the university students consumed supplements according to the suggestions given by their family members. To boost their immune system, a high proportion of Indonesian university students (67.7%) consumed supplements such as multivitamins during the COVID-19 pandemic [27]. Vitamin C was the most consumed supplement owing to its nature in boosting the human immune system [28]. 

More than 90% of the online learning undergraduates did not participate in a weight control program. The finding results from a study conducted among Australian university students revealed that 70% of the respondents were unable to meet the recommended physical activity level during the COVID-19 pandemic as compared to the past two years [7]. This may be due to the temporary closure of fitness centers due to the implementation of the MCO in Malaysia. A nationwide study conducted by Jia et al. [29] in China found out that the COVID-19 lockdown had reduced the physical activity levels and increased the sedentary lifestyle of the studied population.

Based on Table 9, meal skipping was found to be significantly associated (*p* < 0.05) with gender. Females were more likely to skip breakfast intake than males, as they believed breakfast skipping helps to reduce weight [30]. It was reported that females are more concerned about their weight and have more body image distortion than males [31]. Skipping meals is often used as a weight-loss method [30]. However, breakfast skipping is not an effective strategy for losing weight, as it often caused a high intake of energy dense food later in the day [30]. Thus, it will cause weight gain instead of weight reduction.

## 5. Conclusions

In conclusion, the majority of the online learning undergraduates practiced meal skipping and snacking between meals. Health programs emphasizing the importance of regular meal intake and healthy snacks selection could be conducted to promote good eating behaviors among online learning undergraduates. Further studies of larger sample size straddling government and private universities are recommended to understand the eating behaviors of undergraduates after the COVID-19 pandemic.

## Figures and Tables

**Table 1:** Socio-demographic information.

Characteristics	*n* (%)
Gender	
Male	22 (18.03)
Female	100 (81.97)
Ethnicity	
Chinese	116 (95.08)
Indian	5 (4.10)
Malay	1 (0.82)
Age (years)	
20–22	106 (86.89)
23–25	16 (13.11)
Academic year	
Year 1–2	50 (40.98)
Year 3–4	72 (59.02)
Current living arrangement	
Staying with family	101 (82.79)
Staying alone/hostel	21 (17.21)
Height (mean ± SD, cm)	161.69 ± 7.49
Weight (mean ± SD, kg)	59.79 ± 10.37
BMI classification (kg/m^2^)	
Underweight (<18.5)	33 (27.05)
Normal (18.5–24.9)	72 (59.02)
Overweight and Obese (≥25.0)	17 (13.94)

**Table 2:** Frequency of main meals and snack habits.

	Never or Less than Once a Month	1–3 Times per Month	Once a Week	2–3 Days per Week	4–6 Days per Week	Everyday
Main meals						
Breakfast	2 (1.64)	5 (4.10)	5 (4.10)	19 (15.57)	26 (21.31)	65 (53.28)
Lunch	0 (0.00)	0 (0.00)	1 (0.82)	2 (1.64)	12 (9.84)	107 (87.70)
Dinner	1 (0.82)	0 (0.00)	1 (0.82)	1 (0.82)	9 (7.38)	110 (90.16)
Snack between meals						
Morning tea	61 (50.00)	24 (19.67)	12 (9.84)	12 (9.84)	7 (5.74)	6 (4.92)
Afternoon tea	21 (17.21)	31 (25.41)	13 (10.66)	29 (23.77)	23 (18.85)	5 (4.10)
Supper	33 (27.05)	28 (22.95)	14 (11.48)	22 (18.03)	10 (8.20)	15 (12.30)

**Table 3:** Type of foods consumed as snacks by respondents.

No.	Food	*n* (%)	
		Yes	No
1.	Fruits	98 (80.33)	24 (19.67)
2.	Breads	103 (84.43)	19 (15.57)
3.	Malaysian kuih-muih	55 (45.08)	67 (54.92)
4.	Ice-cream	57 (46.72)	65 (53.28)
5.	Snack (packet)	87 (71.31)	35 (28.69)
6.	Biscuit	108 (88.52)	14 (11.48)
7.	Banana fritters	22 (18.03)	100 (81.97)
8.	Nasi lemak	31 (25.41)	91 (74.59)
9.	Chocolate	58 (47.54)	64 (52.46)
10.	Noodles or instant noodles	72 (59.02)	50 (40.98)
11.	Others	19 (15.57)	103 (84.43)
12.	Yogurt	3 (2.46)	119 (97.54)
13.	Nuts	3 (2.46)	119 (97.54)
14.	Seafood	2 (1.64)	120 (98.36)
15.	Porridge/Rice/Rice Ball	1 (0.82)	121 (99.18)
16.	Vegetables	1 (0.82)	121 (99.18)
17.	Fast Food	2 (1.64)	120 (98.36)
18.	Egg	3 (2.46)	119 (97.54)
19.	Cereal/granola/oats/fit bar	6 (4.92)	116 (95.08)
20.	Soup	1 (0.82)	121 (99.18)
21.	Cake	3 (2.46)	119 (97.54)
22.	Spaghetti	1 (0.82)	121 (99.18)

**Table 4:** Types of beverages consumed as snacks by respondents.

No.	Beverage	*n* (%)	
		Yes	No
1.	Tea	71 (58.20)	51 (41.80)
2.	Chocolate malt milk	60 (49.18)	62 (50.82)
3.	Fruit juices	52 (42.62)	70 (57.38)
4.	Milk	81 (66.39)	41 (33.61)
5.	Carbonated drink	34 (27.87)	88 (72.13)
6.	Syrup	5 (4.10)	117 (95.90)
7.	Coffee	46 (37.70)	76 (62.30)
8.	Other beverages	27 (22.13)	95 (77.87)
9.	Multigrain/oat cereal drink/oat milk/cereal drinks	8 (6.56)	114 (93.44)
10.	Milk tea	1 (0.82)	121 (99.18)
11.	Soya milk	2 (1.64)	120 (98.36)
12.	Protein shake	2 (1.64)	120 (98.36)
13.	Cultured Drink	1 (0.82)	121 (99.18)

**Table 5:** Frequency of eating away from home.

Parameter	Response	*n* (%)
Dine in at hawker centers, coffee shops, or other food stalls	Everyday	7 (5.74)
	4–6 days per week	16 (13.11)
	2–3 days per week	19 (15.57)
	Once a week	31 (25.41)
	1–3 times per month	33 (27.05)
	Never or less than once a month	16 (13.11)
Dine in at Western fast-food restaurants	Everyday	0 (0.0)
	4–6 days per week	1 (0.82)
	2–3 days per week	14 (11.48)
	Once a week	16 (13.11)
	1–3 times per month	55 (45.08)
	Never or less than once a month	36 (29.51)

**Table 6:** Dietary practices.

No.	Different Type of Dietary Practices	*n* (%)
1.	Limited intake of high-fat and high-sugar foods	24 (19.67)
2.	Limited intake of high-fat, high-sugar, and red meat foods	15 (12.30)
3.	Limited intake of high-fat foods	3 (2.46)
4.	Consumed specific weight loss diet menu	1 (0.82)
5.	Did not eat according to any special diet menu but reduce food intake to prevent weight gain	20 (16.39)
6.	Were not choosy on the types of food eaten and ate any food available	44 (36.07)
7.	Vegetarians	4 (3.28)
8.	Other	11 (9.02)

**Table 7:** Health supplement intake.

Parameter	Response	*n* (%)
Health supplement intake	Yes	50 (40.98)
	No	72 (59.02)
	Total	122 (100)
Sources of advice on health supplement intake	Parents	25 (50.00)
	Other Family Members (Siblings/ Relatives)	3 (6.00)
	Peers/Friends	2 (4.00)
	Own Decision	14 (28.00)
	Websites/social media	6 (12.00)
	Physicians	0 (0.0)
	Total	50 (100)

**Table 8:** Weight control program participation.

Parameter	Response	*n* (%)
Participation in weight control program	Yes	5 (4.1)
	No	117 (95.90)
	Total	122 (100.0)
Types of programs	Gyms	2 (40.00)
	Sport	2 (40.00)
	Yoga	1 (20.00)
	Aerobic	0 (0.0)
	Total	5 (100)
Sources of advice on weight change program participation	Parents	2 (40.00)
	Own Decision	1 (20.00)
	Websites/social media	2 (40.00)
	Peers/Friends	0 (0.0)
	Other Family Members (Siblings/Relatives)	0 (0.0)
	Physicians	0 (0.0)
	Total	5 (100)

**Table 9:** Association between sociodemographic variables and meal skipping behaviors.

Sociodemographic	Meal Skipping, *n* (%) *	
	Yes	No
Gender		
Male	6 (27.27)	16 (72.73)
Female	58 (58.00)	42 (42.00)
	*χ*^2^ = 6.83, *p* = 0.01	
Age		
20–22	55 (51.89)	51 (48.11)
23–25	9 (56.25)	7 (43.75)
	*χ*^2^ = 0.11, *p* = 0.75	
Academic year		
Year 1 and 2	29 (58.00)	21 (42.00)
Year 3 and 4	35 (48.61)	37 (51.39)
	*χ*^2^ = 1.04, *p* = 0.31	
Current living arrangement		
Staying with Family	50 (49.50)	51 (50.50)
Staying alone	14 (66.67)	7 (33.33)
	*χ*^2^ = 2.05, *p* = 0.15	
BMI		
Underweight	18 (54.55)	15 (45.45)
Normal weight	34 (47.22)	38 (52.78)
Overweight and obese	12 (70.59)	5 (29.41)
	*χ*^2^ = 3.09, *p* = 0.21	

* Chi-square analysis (*χ*^2^) with significance at *p* < 0.05.
